# SVAD: A genetic database curates non-ischemic sudden cardiac death-associated variants

**DOI:** 10.1371/journal.pone.0237731

**Published:** 2020-08-19

**Authors:** Wei-Chih Huang, Hsin-Tzu Huang, Po-Yuan Chen, Wei-Chi Wang, Tai-Ming Ko, Sirjana Shrestha, Chi-Dung Yang, Chun-San Tai, Men-Yee Chiew, Yu-Pao Chou, Yu-Feng Hu, Hsien-Da Huang

**Affiliations:** 1 Department of Biological Science and Technology, National Chiao Tung University, Hsinchu, Taiwan, R.O.C; 2 Institute of Bioinformatics and Systems Biology, National Chiao Tung University, Hsinchu, Taiwan, R.O.C; 3 Industrial Development Graduate Program of College of Biological Science and Technology, National Chiao Tung University, Hsinchu, Taiwan, R.O.C; 4 Institute of Biomedical Sciences, Academia Sinica, Taipei, Taiwan, R.O.C; 5 Graduate Institute of Integrated Medicine, College of Chinese Medicine, China Medical University, Taichung, Taiwan, R.O.C; 6 Warshel Institute for Computational Biology, The Chinese University of Hong Kong, Shenzhen, China; 7 School of Life and Health Sciences, The Chinese University of Hong Kong, Shenzhen, China; 8 Institute of Molecular Medicine and Bioengineering, National Chiao Tung University, Hsinchu, Taiwan, R.O.C; 9 Division of Cardiology, Department of Medicine, Taipei Veterans General Hospital, Taipei, Taiwan, R.O.C; 10 Institute of Clinical Medicine, and Cardiovascular Research Center, National Yang-Ming University, Taipei, Taiwan, R.O.C; Heart and Diabetes Center NRW, UNiversity Hospital of the Ruhr-University Bochum, GERMANY

## Abstract

Sudden cardiac death (SCD) is an important cause of mortality worldwide. It accounts for approximately half of all deaths from cardiovascular disease. While coronary artery disease and acute myocardial infarction account for the majority of SCD in the elderly population, inherited cardiac diseases (inherited CDs) comprise a substantial proportion of younger SCD victims with a significant genetic component. Currently, the use of next-generation sequencing enables the rapid analysis to investigate relationships between genetic variants and inherited CDs causing SCD. Genetic contribution to risk has been considered an alternate predictor of SCD. In the past years, large numbers of SCD susceptibility variants were reported, but these results are scattered in numerous publications. Here, we present the SCD-associated Variants Annotation Database (SVAD) to facilitate the interpretation of variants and to meet the needs of data integration. SVAD contains data from a broad screening of scientific literature. It was constructed to provide a comprehensive collection of genetic variants along with integrated information regarding their effects. At present, SVAD has accumulated 2,292 entries within 1,239 variants by manually surveying pertinent literature, and approximately one-third of the collected variants are pathogenic/likely-pathogenic following the ACMG guidelines. To the best of our knowledge, SVAD is the most comprehensive database that can provide integrated information on the associated variants in various types of inherited CDs. SVAD represents a valuable source of variant information based on scientific literature and benefits clinicians and researchers, and it is now available on http://svad.mbc.nctu.edu.tw/.

## Introduction

Sudden cardiac death (SCD) is defined as the unexpected death occurring within 1 h from the onset of symptoms in a subject with no known prior fatal condition [1, 2]. SCD is a major public health issue, accounting for approximately half of all deaths from cardiovascular diseases [3, 4]. In East Asia, particularly in China [3, 5], Japan [3, 6], and Taiwan [3, 7], the incidence of SCD per 100,000 cases per year was 41.8, 14.9, and 28.4, respectively. By contrast, the annual incidence of SCD ranges from 50 to 100 in the USA and Europe, which is relatively higher than that in East Asia [3, 8]. Sudden cardiac death in the young is a devastating event. The incidence of SCD in the younger population (< 40 years) is 1.8–2.8/100,000 individuals per year, and it makes up a significant proportion of the mortality in this age group [2, 9–16]. While coronary artery disease and acute myocardial infarction account for the majority of SCD in the elderly population, inherited cardiac diseases (inherited CDs) comprise a substantial proportion of younger SCD victims with a significant genetic component [14, 17–23]. In half of young SCD victims, the etiology has been reported to be inherited CDs [2, 24]. Inherited cardiac diseases include cardiomyopathies (e.g., hypertrophic cardiomyopathy (HCM), dilated and restrictive cardiomyopathies, arrhythmogenic right ventricular cardiomyopathy (ARVC) and left ventricular non-compaction) and channelopathies (e.g., long QT syndrome (LQTS), Brugada syndrome (BrS), catecholaminergic polymorphic ventricular tachycardia (CPVT), idiopathic ventricular fibrillation and short-QT syndrome). Cardiomyopathy begins to dominate in older children and young adults [25–27]. HCM remains the most common structural cause of SCD in the young [28], but ARVC might cause SCD in up to 25% in some countries [26]. In the United States, the most common causes of SCD in the young are HCM (~40% to 50%), arrhythmias (~20%), other cardiomyopathies (~10%), and others [29].

Identifying the genetic factors predisposing to SCD is important, and genetic biomarkers are considered alternate predictors because the majority of patients hold preserved cardiac function [4]. Owing to the recent developments in sequencing, the use of next-generation sequencing (NGS) enables the rapid analysis of many genes responsible for inherited CDs [30]. NGS allows a fast and cost-effective approach for genetic screening of a large set of genes. It is rapidly applied to clinical practice and allows scientists to investigate the genetic variants contributing to specific phenotypes when combined with large-scale annotated genetic databases [31]. At present, clinical research aims to exploit the potential of genetic variation as risk predictors or biomarkers to prevent SCD based on the clinical stages of patients and their relatives [32–36]. Therefore, genetic testing is considered practicable in early diagnosis, prognostic stratification, and therapeutic interventions [36, 37]. In a 5.5-year follow-up study, it showed that the overall diagnostic yield of inherited CDs in 304 SCD families was 47%, and most diagnoses identified in the relatives were related to the diagnosis in the proband [38]. In 2015, the American College of Medical Genetics together with the Association of Molecular Pathology (ACMG-AMP) published guidelines to set standards on determining the pathogenicity of variants [39]. More importantly, a committee of the European Society of Human Genetics (ESHG) developed recommendations on how to integrate genetic testing into multidisciplinary management of SCD [40]. However, the clinical interpretation of identified variants remains a challenge because of scattered or insufficient evidence supporting their pathogenic effects.

In the past years, numerous studies aiming to explore the genetic susceptibility of non-ischemic SCD were published. Large numbers of susceptibility variants and genes have been reported to be disease-associated. However, these results are scattered in numerous publications and are sometimes inconsistent because of differences in the allele frequency among different populations [41]. A centralized information repository for a comprehensive and well-organized collection of genetic data from multiple published studies is urgently needed to provide lots of evidence to clarify the genetic predisposition to non-ischemic SCD. Here, we present the SCD-associated Variants Annotation Database (SVAD) to facilitate the interpretation of variants and to fulfill the needs of data integration for non-ischemic SCD caused by cardiomyopathies and channelopathies. In the present work, literature that mentioned the associations between genetic variants and one of the inherited CDs (HCM, ARVC, LQTS, BrS and CPVT) causing non-ischemic SCD was searched and collected. From the selected papers, we manually collected details on the type of variation and information. We also provided data from *in silico* prediction methods to aid the interpretation of variations with amino acid change; thus, we can classify whether the published variants are deleterious or not. SVAD currently contains approximately 2,300 entries within 1,239 distinct variants of 12 key genes associated with non-ischemic SCD, which were referenced from 232 published studies. In general, SVAD is designed to help unveil the genetic basis of SCD caused by inherited CDs.

## Materials and methods

### Literature collection and data integration

In our database, the associations between non-ischemic SCD caused by inherited CDs and genetic variants were provided and derived from full-text literature reading with manual curation of these genetic studies. It was presently focused on five types of inherited CD (i.e., HCM, ARVC, LQTS, BrS and CPVT) causing non-ischemic SCD, and associated literature was searched in the Entrez PubMed (http://www.ncbi.nih.gov/pubmed) using “hypertrophic cardiomyopathy”, “arrhythmogenic right ventricular cardiomyopathy”, “long QT syndrome”, “Brugada syndrome”, “catecholaminergic polymorphic ventricular tachycardia” and “Date—Publication from 2011/01/01 to 2018/03/31” as search terms. A total of 1,077 articles with available pdf files were collected. Articles and reviews mentioned about associations of genetic variation and SCD were kept for further reading by the manual screening of these publications. Initially, 4,033 entries were collected from 368 articles. Next, the collected variants located in the coding sequence (CDS) of 12 important genes related to HCM, ARVC, LQTS, BrS or CPVT (i.e., *MYBPC3*, *MYH7*, *DSP*, *PKP2*, *CACNA1C*, *CACNB2*, *KCNE1*, *KCNE2*, *KCNH2*, *KCNQ1*, *SCN5A*, and *RYR2*) were extracted for further curation and data integration.

Integrated variant information includes: associated inherited CD; located gene; chromosomal location (human reference genome version GRCh37); reference transcript ID in RefSeq; amino acid change and coding DNA change based on the reference transcript sequence; types of alteration (nonsense, missense, insertion-deletion or synonymous); the Human Genome Variation Society (HGVS) nomenclature of cDNA or protein sequence; corresponding SNP ID in dbSNP (if available) [42]; ethnicity and number of cases in each reference literature; PubMed ID of reference literature; *in silico* functional prediction for variants with amino acid change using the Combined Annotation Dependent Depletion (CADD) tool [43]; interpretation in ClinVar (if available) [44]; reported classification following the ACMG guideline (e.g., pathogenic, likely-pathogenic, variant of uncertain significance, likely-benign or benign) from VarSome [45]; allele frequency of each variant in different populations retrieved from the ExAC database [46], the 1000 Genome Project [47] and the Taiwan Biobank [48] (**Table 1**).

**Table 1:** Integrated features in SVAD. The features were integrated from various public databases and were curated for presenting in browse page and result page.

Features	Descriptions
Accession ID	Each entry is assigned an SVAD Accession ID, e.g. SVAD0389
Reported classification	Variants classification following the ACMG guideline is retrieved from Varsome
Disease	Associated inherited cardiac diseases for each entry
Gene	The located gene of a variant
Chromosome & Location (GRCh37)	Located chromosomal location of a variant using human reference genome GRCh37 version
REF allele	Reference allele of a variant
ALT allele	Alternative allele of a variant
SNP ID	The rs number used in dbSNP
Transcript ID	The corresponding transcript of a variant with a RefSeq ID will be used for the HGVS nomenclature
Nucleotide change	cDNA nucleotide change of a variant in the corresponding transcript following the HGVS nomenclature
Amino acid change	Amino acid change of a variant in the corresponding transcript following the HGVS nomenclature
Alteration type	Genetic alteration of a variant is indicated, e.g. missense, nonsense, indel and synonymous
PubMed ID	Article ID of reference literature for each entry from PubMed
Population	The population of recruited individuals in a reference literature
Case number	Total number of individuals who suffered non-ischemic SCD in a reference literature
Control number	Total number of individuals who did not suffer non-ischemic SCD in a reference literature
ClinVar	Variant interpretation retrieved from ClinVar
CADD: SIFT prediction	Functional prediction of a variant by CADD (SIFT part)
CADD: SIFT score	Prediction score of CADD (SIFT part)
CADD: Polyphen prediction	Functional prediction of a variant by CADD (PolyPhen part)
CADD: Polyphen score	Prediction score of CADD (PolyPhen part)
Allele frequency in dbSNP	Global minor allele frequency (GMAF) of variants retrieved from dbSNP
Allele frequency in 1000 Genomes	Allele frequency of variants for five populations retrieved from 1000 Genomes
Allele frequency in ExAC	Allele frequency of variants for five populations retrieved from ExAC database
Allele frequency in Taiwan Biobank	Allele frequency and genotypic frequency of variants for Taiwanese retrieved from Taiwan Biobank

Independent studies usually describe genetic variation using amino acid changes or cDNA changes in a gene without indicating the reference transcript nor clearly describing the chromosomal position of the gene. To overcome this problem, all variants were described according to the HGVS nomenclature guidelines [49], the nomenclatures for variants were curated by dbSNP, ClinVar, and VarSome, and chromosomal location of variants following human reference genome version GRCh37 was used. Functional prediction by CADD can serve to improve the interpretation of genetic variants. Relationships among genetic variants and phenotypes with supporting evidence are retrieved from ClinVar. Classification of variants following the ACMG guideline can help researchers evaluate pathogenicity. Additionally, the ethnicity of cases in reference literature and allele frequency of variants in different populations were presented because the distribution of inherited CD-associated variants might be different in varied populations [41].

## Results

### SVAD data statistics

**Fig 1:**
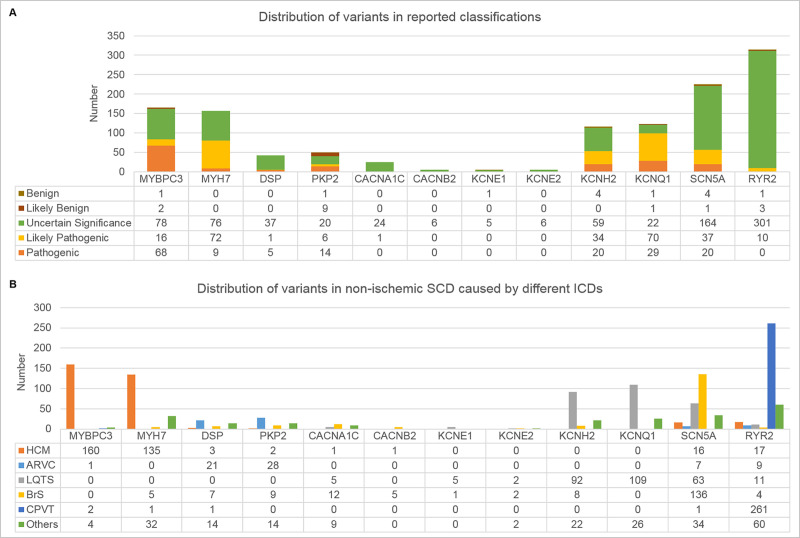
The number of variants (A) in different reported classifications or (B) in different inherited CDs causing non-ischemic SCD. (A) A total of 1,239 variants located in the CDS of 12 key genes associated with five types of inherited CD (HCM, ARVC, LQTS, BrS and CPVT) were curated. Variants of uncertain significance accounted for 64.4% of curated variations. Pathogenic and likely-pathogenic variants were 13.3% and 19.9%, respectively. A high proportion of pathogenic variants was observed in *MYBPC3*. (B) In HCM, a high proportion of non-ischemic SCD-associated variants of *MYBPC3* and *MYH7* was observed and some variants of *SCN5A* and *RYR2* were shown. In ARVC, variants of *PKP2* and *DSP* accounted for high proportion. In LQTS, most non-ischemic SCD-associated variants were derived from *KCNQ1*, *KCNH2* and *SCN5A*. In BrS, variants of SCN5A accounted for the majority. In CPVT, the majority of non-ischemic SCD-variants belonged to *RYR2*. HCM: Hypertrophic cardiomyopathy; ARVC: Arrhythmogenic right ventricular cardiomyopathy; LQTS: Long QT syndrome; BrS: Brugada syndrome; CPVT: Catecholaminergic polymorphic ventricular tachycardia.

**Fig 2:**
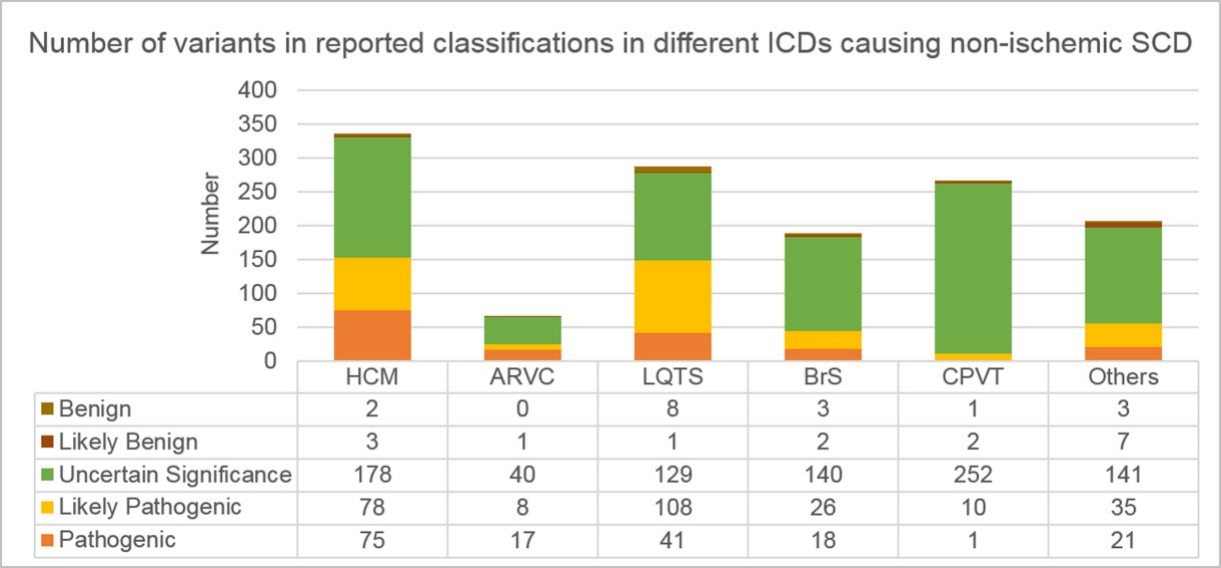
Distribution of reported classifications of variants in different inherited CDs causing non-ischemic SCD. Variants of uncertain significance accounted for the majority in these inherited CDs. The percentage of pathogenic/likely-pathogenic variants was high in LQTS and HCM, accounting for 51.9% and 45.5%, respectively. HCM: Hypertrophic cardiomyopathy; ARVC: Arrhythmogenic right ventricular cardiomyopathy; LQTS: Long QT syndrome; BrS: Brugada syndrome; CPVT: Catecholaminergic polymorphic ventricular tachycardia.

SVAD is now available at http://svad.mbc.nctu.edu.tw/ and will be updated annually and the updates applied in the database are reported in the “Latest news” archive on the homepage. In the current release of SVAD, a total of 2,292 entries within 1,239 variants located in the CDS of 12 key inherited CD-related genes were extracted from 232 articles (**Table 2**). The key genes were selected according to our ICDscreening panel (unpublished), and this panel included only the established genes with significant clinical impact, high prevalence, and clear and relevant pathogenetic mechanisms. Most collected variations were missense type, accounting for 83.2% (1,031/1,239); percentage of nonsense, indel and synonymous variations were 5.7%, 9.5% and 1.5%, respectively (**Table 2**). Top 5 number of entries for genes were *RYR2* (970/2,292, 42.3%), *SCN5A* (306/2,292, 13.4%), *MYH7* (263/2,292, 11.5%), *MYBPC3* (251/2,292, 11.0%) and *KCNQ1* (201/2,292, 8.8%), and top 5 number of collected variants for genes were *RYR2* (315/1,239, 25.4%), *SCN5A* (226/1,239, 18.2%), *MYBPC3* (165/1,239, 13.3%), *MYH7* (157/1,239, 12.7%) and *KCNQ1* (123/1,239, 9.9%). For *MYBPC3* and *PKP2*, their indel variations occupied above 20% of their total variations. The number of variants with different reported classifications for each gene were shown in **Fig 1A**. Variants of uncertain significance accounted for 64.4% of curated variations. Pathogenic and likely-pathogenic variants were 13.3% and 19.9%, respectively. The percentage of pathogenic variants and likely-pathogenic variants was higher in *KCNQ1*, *MYH7*, *MYBPC3*, *KCNH2*, and *PKP2* than in others. A high proportion of pathogenic variants was observed in *MYBPC3*. Distribution of variants associated with various inherited CDs were shown in **Fig 1B.** Among these variants, most HCM-associated variants were derived from *MYBPC3* and *MYH7*; most ARVC-associated variants were derived from *PKP2* and *DSP*; most LQTS-associated variants were derived from *KCNQ1*, *KCNH2* and *SCN5A*; most BrS-associated variants were derived from *SCN5A* and *CACNA1C*; most CPVT-associated variants were derived from *RYR2*. The percentage of pathogenic/likely-pathogenic variants was high in LQTS and HCM, accounting for 51.9% and 45.5%, respectively (**Fig 2**). Furthermore, 413 out of 1,239 variants, approximately one-third of collected variants, were pathogenic/likely-pathogenic in SVAD. Some pathogenic/likely-pathogenic variants were mentioned many times in literature, e.g. p.Arg176Gln variation of *RYR2* (14 times), p.Arg719Trp variation of *MYH7* (10 times) and p.Ile4867Met variation of *RYR2* (9 times). Thus, once a subject carries these pathogenic/likely-pathogenic variants, regardless of whether he has obvious symptoms of inherited CD, is likely to be at high risk of non-ischemic SCD. The number of variants associated with various inherited CDs in different populations was listed in **Table 3**. Indeed, the majority of research results in non-ischemic SCD focus on the Caucasian population, accounting for 42.8%. However, it was indicated that the disease-associated variants with high incidence could vary in different populations [41]. In our opinion, the evaluation of variants for potential pathogenicity in different populations should depend on the allele frequency data derived from the corresponding population, not on the general population data.

**Table 2:** The number of collected articles, curated entries and variants in 12 selected genes reported in SVAD.

Gene	Associated inherited CD	No. of articles	No. of entries	No. of variants	No. of alterations			
					Missense	Nonsense	Indel	Synonymous
*MYBPC3*	HCM	35	251	165	95	31	38	1
*MYH7*		35	263	157	152	0	4	1
*DSP*	ARVC	16	48	43	37	4	2	0
*PKP2*		19	62	50	23	8	13	6
*RYR2*	CPVT	91	970	315	300	1	14	0
*SCN5A*	LQTS & BrS	69	306	226	195	9	15	7
*CACNA1C*		13	37	25	23	0	2	0
*CACNB2*		3	6	6	6	0	0	0
*KCNE1*		10	10	6	6	0	0	0
*KCNE2*		5	6	6	6	0	0	0
*KCNH2*		41	132	117	91	7	16	3
*KCNQ1*		38	201	123	97	11	14	1
Total		2,292	322	1,239	1,031	71	118	19

Inherited CD: inherited cardiac disease; Indel: Insertion-deletion; HCM: Hypertrophic cardiomyopathy; ARVC: Arrhythmogenic right ventricular cardiomyopathy; CPVT: Catecholaminergic polymorphic ventricular tachycardia; LQTS: Long QT syndrome; BrS: Brugada syndrome.

**Table 3:** Distribution of curated variants associated with various inherited CDs causing non-ischemic SCD in different populations.

Disease	Population	No. of			
		Entries	Variants	Genes^a^	Articles
All	All	2,292	1,239	12	362
Hypertrophic cardiomyopathy (HCM)	All	502	336	8	44
	Caucasian	369	266	6	25
	Asian	99	84	6	11
	African	8	8	2	3
	Others	26	20	2	7
Arrhythmogenic right ventricular cardiomyopathy (ARVC)	All	68	66	5	14
	Caucasian	51	50	5	11
	Asian	10	10	1	1
	African	0	0	0	0
	Others	7	7	3	2
Long QT syndrome (LQTS)	All	387	287	7	62
	Caucasian	198	164	6	33
	Asian	112	88	5	14
	African	5	4	2	4
	Others	72	68	5	13
Brugada syndrome (BrS)	All	237	189	10	33
	Caucasian	207	165	10	17
	Asian	12	12	3	8
	African	5	5	1	1
	Others	13	12	2	7
Catecholaminergic polymorphic ventricular tachycardia (CPVT)	All	853	266	5	67
	Caucasian	198	135	5	37
	Asian	98	69	1	8
	African	0	0	0	0
	Others	557	172	1	23
Combined inherited CDs or inherited CD not in the above	All	245	207	10	47
	Caucasian	191	165	10	32
	Asian	34	30	7	12
	African	3	2	2	3
	Others	17	14	2	3

^a^It includes 12 key genes (i.e., *MYBPC3*, *MYH7*, *DSP*, *PKP2*, *CACNA1C*, *CACNB2*, *KCNE1*, *KCNE2*, *KCNH2*, *KCNQ1*, *SCN5A*, and *RYR2*) in five types of inherited CD in our ICDscreening panel (unpublished).

### SVAD Web interface

**Fig 3:**
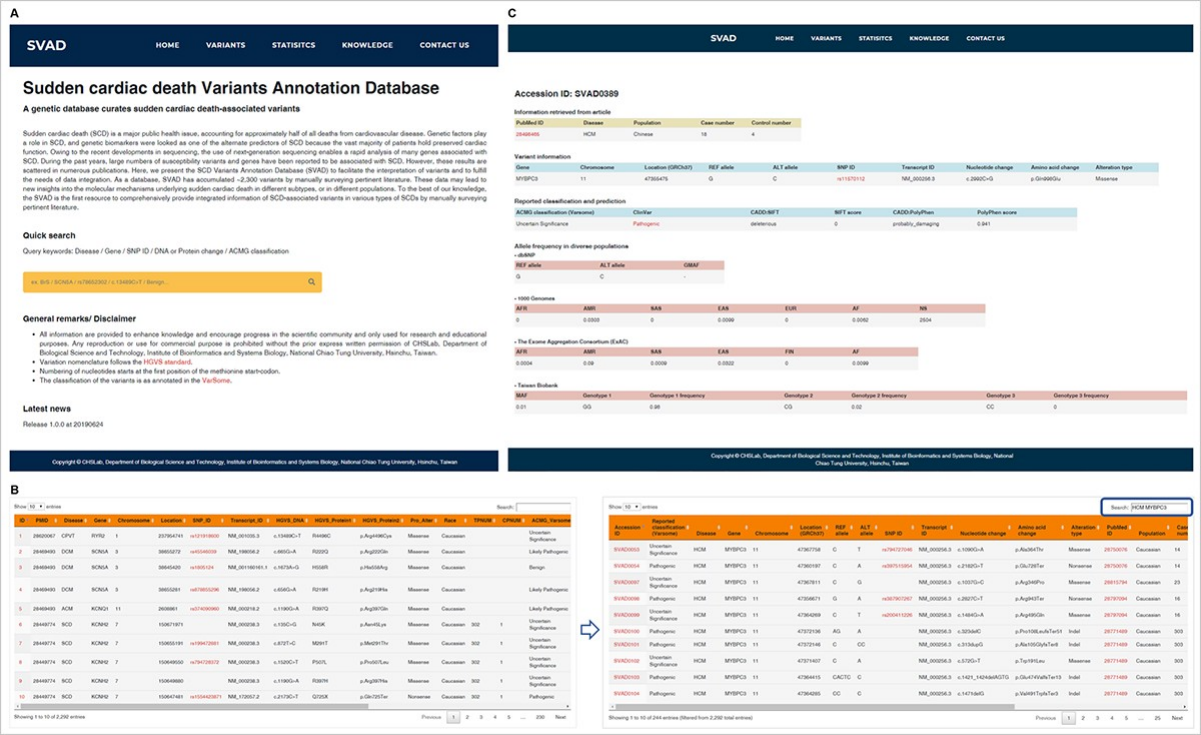
The web interface of SVAD. (A) Users can use keywords such as type of inherited CD abbreviation, gene name, SNP ID, nucleotide/amino acid change or reported classification to query genetic variants of interest. (B) In the browse page, users can change the ordering of results by clicking each column name of the table. At the upper-right search box in the browse page, users can input keywords to quickly query the variants of interest. (C) Detailed information of each variant is shown when clicking on the accession ID.

SVAD provides a user-friendly web interface (**Fig 3**). It presents several search functions for users to facilitate the access of inherited CD-associated variants, including search by diseases, genes, variants, and reported classification (**Fig 3A**). Upon browsing, every 10 results are shown on each page, thereby providing users the opportunity to know whether a variant has been identified and enabling users to search for inherited CD-associated or pathogenic variants quickly and conveniently. Users can input keywords at the upper-right search box in browse page to quickly query for the variants of interest. Keywords should be separated by a whitespace character. For example, only the variations of *MYBPC3* in HCM would be shown when the keywords “HCM MYBPC3” were inputted (**Fig 3B**). Here, a result page is designed to describe variants, where each variant is assigned an SVAD accession ID. The detailed genetic information of each variant, including population frequency, is shown in the result page when the user clicks the SVAD accession ID (**Fig 3C**).

### Comparison with existing resources

To the best of our knowledge, SVAD is the most comprehensive database providing integrated information of variants associated with non-ischemic SCD by manually surveying pertinent literature. In SVAD, integrated information about variants associated with non-ischemic SCD is freely available, such as associated inherited CD, reported classification, clinical significance, predicted functional change, and population allele frequencies. We believe that the comprehensive collection of variant information in SCDs could valuably facilitate the interpretation of genetic data and complement the unmet clinical needs.

As compared to the ARVD/C Genetic Variants Database, which is the only database of inherited CD with variant information, it provided genetic information on only ARVC-related genes and their variants [50]. A total of 1,426 variants located in 12 genes were retrieved from 172 articles. Although it included much information to present a comprehensive view for ARVC, it did not focus on relationships among variants and non-ischemic SCD and did not take allele frequency in various populations and clinical evidence from ClinVar into account. Information of these two factors are important to variant classification. Several other databases provide information on disease-associated variations, but not specific for inherited CD or non-ischemic SCD. The Human Gene Mutation Database (HGMD) constitutes a comprehensive collection of genetic variants that are causally associated with a phenotype or disease [51]. However, there is limited and out-of-date information in the public version of the HGMD [52, 53]. Users must purchase a license of HGMD®Professional to obtain detailed and integrated information. ClinVar is a freely accessible archive of human genetic variants and interpretations of their relationships to disease and it becomes a valuable resource for clinical genetics research. Interpretations of variants are manually reviewed and curated by experts, but it takes much time for this task. There are lots of variants and diseases included in ClinVar, so that interpretations of the variants associated with non-ischemic SCD from the latest research cannot be expected to update promptly. Additionally, some problems in ClinVar are mentioned, such as classification discrepancies between ClinVar and laboratories [54, 55], and out‐of‐date interpretations of some variants [54]. VarSome integrates massive information from multiple databases to provide a comprehensive view for human variation and enables the community to freely and easily share knowledge on them [45]. If a user wants to realize details of the variants that he has collected, VarSome is a very convenient search engine and powerful database to provide information. Nevertheless, he cannot directly browse organized information about the variants associated with non-ischemic SCD. Another concern is that annotations of relationships between diseases and variants reported by users’ contribution could be insufficient owing to minor usage of users studied in non-ischemic SCD.

## Discussions

### Summary

This work devises a frequently updated database called SVAD by continuously surveying pertinent research articles to make the database become a major repository for linking associations of non-ischemic SCD and human genetic variants. A total of 2,292 entries within 1,239 associated genetic variants in 12 key genes were included from 232 articles. The key genes were selected according to our ICDscreening panel (unpublished), and this panel included only the established genes with significant clinical impact, high prevalence, and clear and relevant pathogenetic mechanisms. The SVAD currently represents the most comprehensive source of information regarding non-ischemic SCD-associated variants, thereby providing an overview of known genetic information. To investigate the relationships between disease and variants, information on the clinical significance of variants was retrieved from ClinVar. For elucidating the biological meanings of the reported nonsynonymous mutations, the CADD tool was implemented to provide *in silico* functional prediction of variants. To clarify whether a variation is a rare variant and to improve interpretation of variants in ethnically diverse populations, allele frequency data from the 1000 Genomes, ExAC and Taiwan Biobank is included. The pathogenicity of each genetic variant could be accurately emphasized when these data are integrated, and it may lead to new insights into the molecular mechanisms underlying inherited CDs in different subtypes or populations. This comprehensive collection of genetic data about non-ischemic SCD caused by inherited CD represents a valuable source of integrated information on the spectrum of disease-associated variations, thereby benefiting clinicians and researchers. Researchers and clinicians can rapidly verify whether the variation of interest has been published and obtain the supporting evidence of pathogenicity.

### Limitations

There are some limitations to this work. At this first release, we focus on the 12 key genes, which are related to the five types of inherited CD (HCM, ARVC, LQTS, BrS, and CPVT) causing non-ischemic SCD, with significant clinical impact, high prevalence, and clear and relevant pathogenetic mechanisms. We are preparing for collecting articles and integrating information on the other related genes of these inherited CDs, and it is expected to complete at the next release. For the novel investigated genes, they are not included because their clinical significances, prevalence, and mechanisms might be unclear. We will evaluate the strength of supporting evidence of variants and provide an indicator for inclusion to describe their associations and importance in a further update. The inclusion of the five inherited CDs is not a constraint for our works. We will gradually link the associations of variants to other inherited CDs causing non-ischemic SCD, such as short-QT syndrome and dilated cardiomyopathy, in the next years. Presently, associations of the five types of inherited CD and variants were retrieved from the literature published from 2011 to 2018. The collection and curation of associations that appeared in literature published in previous years will be complemented as soon as possible. Additionally, a large number of related genes and a high proportion of family-specific variations in inherited CDs make it a complicated disorder [19]. Although cosegregation data is important for evaluating the pathogenicity strength of variants, it is dispersed in literature [56]. It is a complicated task and we will spend much time and effort to systematically collect, retrieve, and validate cosegregation data in numerous publications.

### Perspective works

Three prospective works need to be performed in the near future. First, future work involving the proposed database should include more data about associations of genetic variants and other types of inherited CD. Second, the predicted functional status and pathogenicity of the probable inherited CD-associated variants will be regularly updated and revised. Third, to address the heterogeneity of studies, the impacts of varying allele frequency of variants in various populations should be evaluated. Bias is observed in the genetic studies of disease-associated variants because the majority of them are discovered in Caucasian populations [47, 57, 58]. Our collected data also show a similar phenomenon (**Table 3**). It was also indicated that the risk of SCD was also possibly influenced by race and ethnicity [59]. Besides, following the ACMG guideline, a variation is considered “benign” when its allele frequency is equal to or higher than 5% in a population [39]. Nevertheless, the discrepancy is observed in the allele frequency of variants in other understudied ethnically diverse populations [41]. Realizing differences of allele frequencies across populations could provide new insights into the pathogenicity of some specific variants, which could help in developing a scoring method for evaluating the influence of variants in various populations. In response to the rapid growth of genetic data, natural language processing techniques will be implemented to effectively screen a large number of studies to collect information about types of inherited CD, population, and the number of included samples, experimental methods, the panel of genes, disease-associated variants and cosegregation data. The established method will reduce the effort for the curators. Collecting evidence of molecular regulation from omics data is another direction to figure out the big picture of non-ischemic SCD.

## Supporting information

S1 File The list of curated genetic variants and PMID of available articles in SVAD. (TXT)
